# Twelve Tips for Teaching with Virtual Learning Platforms

**DOI:** 10.15694/mep.2021.000072.1

**Published:** 2021-03-16

**Authors:** Kheyandra Lewis, Zia Bismilla, Nicholas Kuzma, Jennifer O'Toole, Sharon Calaman

**Affiliations:** 1 Drexel University College of Medicine/St. Christopher's Hospital for Children; 2 University of Toronto/Hospital for Sick Children; 3 University of Cincinnati College of Medicine/Cincinnati Children's Hospital Medical Center; 4 New York University School of Medicine/New York University Langone Health/Hassenfeld Children's Hospital

**Keywords:** Virtual Education, Medical Education, Faculty Development

## Abstract

This article was migrated. The article was marked as recommended.

Met with the challenge of physical distancing during the escalation of the COVID-19 pandemic, medical educators rapidly pivoted their educational repertoires to virtual learning platforms. While selection and utilization of virtual platforms may vary amongst medical educators, elements of evidence-based educational theories, collaborative learning, and learner engagement are essential to the success of learning for any format. In this piece we outline 12 tips for virtual learning, drawing on concepts from available literature and our collective experience as medical educators. As virtual learning platforms continue to evolve, medical educators should leverage different modalities, without compromising the fundamental elements and theories that promote learner success.

## Introduction

As the COVID-19 pandemic escalated in the spring of 2020, medical educators had to quickly adapt their educational practices to allow for physical distancing, while maintaining the delivery of evidence-based educational methodologies that engaged learners. To achieve their educational missions, many medical educators shifted to virtual learning platforms. While virtual learning platforms have been in use for quite some time, medical educators are now learning how to use them on a larger scale and for an extended duration of time.

The transition to teaching on virtual platforms can be overwhelming. Rather than focusing on the technology, however, it is essential to maintain a focus that is learner-centered. A technology-centered approach builds a curriculum around the capabilities of the available technological resources. A learner-centered approach carefully considers how the available technology can provide opportunities for active learning and advance the learner’s understanding of material. There is no “super technology” that is the best for virtual education. Learner satisfaction is related to attention from instructors and the delivery of content that meets their needs. As stated by Clark, “Emerging technologies are alluring because of their novelty, but this should not be confused with their effectiveness as vehicles for learning. In fact, the mere exposure to technology, new or old, does not confer any particular educational benefits. The way a given technology is incorporated into the instructional method is more important than the capabilities of the technology itself” ( Clark, 1994). The 12 tips outlined below focus on strategies to deliver virtual learning to promote teacher satisfaction and maximize learner experience and knowledge retention.

Consider the tips as a BOX:

1. Prepare and plan in advance
2. Establish session etiquette
3. Build community
4. Enhance dialogue and engagement
5. Focus on learner-centered goals and objectives
6. Optimize active learning
7. Consider cognitive load
8. Incorporate cooperative group learning
9. Invite learner feedback
10. Allow learners time to reflect
11. Acknowledge instructors’ need to reflect
12. Incorporate ongoing faculty development

## Tip 1: Prepare and plan in advance

Every virtual learning platforms has its own distinctive capabilities such as the number of learners accommodated, breakout rooms, file sharing, polling, chats, screen sharing, and audio-visual configurations. Familiarize yourself with the key features of your chosen platform. Conduct video and audio testing of your equipment ahead of time to guarantee the session runs smoothly. Maximize the appearance of your screen by enabling high definition video, having a solid colored wall behind you or masking the appearance of your background using a virtual background feature, and ensuring front or overhead lighting to highlight your face. Consider security and confidentiality issues if sensitive information will be presented thinking about where you are situated and who may see or hear you. For sensitive or confidential content, set up a new meeting ID and password for every session, share the link directly with learners via an institutional email address and remind them it is for individual use only. If recording, verify that all learners have provided consent and store the recording in a secure location. If you plan to share your screen, be certain your desktop is ready to be viewed and essential documents or files are accessible. Close non-essential windows to maximize the speed of the virtual learning software.

Learners must also gain confidence and competence in the technology of virtual learning. Provide clear instructions to learners in advance of the session, including links to virtual learning platforms, participant identification numbers, and internet browser requirements. Ask learners to test software, internet capabilities, cameras, and audio before the session to avoid unanticipated issues. Instruct learners to log on early to trouble shoot any last-minute issues so that the session can start on time. To assure inclusivity, consider different time zones when scheduling your session, or consider recording your session for learners to view at a different time.

## Tip 2: Establish session etiquette

Once all learners have joined your session, conduct a brief audiovisual check, advise about possible disconnections, and provide instruction on how to reconnect if one occurs. Consider how you would like learners to display their names and encourage re-naming when necessary. Orient learners to features of your virtual learning platform that will be utilized during the session, such as the use of break out rooms and “raise hand” functions. Set ground rules for the session, for example, establish the expectation that cameras remain on to enhance learner presence or that learners should remain on mute when not speaking to eliminate distractions and background noise. If you plan to use the chat function, ensure someone is assigned to read and monitor the chat and can inform you of content that requires your attention.

## Tip 3: Build a sense of community

Virtual learning can feel impersonal and isolating. To avoid this, it is essential to cultivate a sense of community in the virtual learning environment. This is particularly important when learners will be working together for multiple sessions. Community is built in virtual learning environments through sharing personal experiences and values, developing mutual awareness and respect, and creating a sense of group commitment ( Garrison, Anderson and Archer, 1999). Instructors should consider building time into their educational delivery to foster relationships through the use of introductions and icebreakers, addressing students by name, sharing personal stories or professional experiences, encouraging diverse points of view, and providing opportunities where students can communicate with each other ( Fiock, 2020). As the instructor, try to convey warmth and presence by making “eye contact.” Look directly at your camera when speaking whenever possible. Incorporating these exercises encourages communication and promotes interactivity, contributing to the development of a learning community.

## Tip 4: Enhance dialogue and engagement

Ongoing engagement between learners and instructors is vital when using virtual learning platforms to maintain the integrity of the learning environment and assess knowledge acquisition. The instructor must be sure to create a learning environment that promotes dialogue amongst learners, as well as between the instructor and learners. As described in Tip 2, setting ground rules such as camera use, lays the foundation for maximizing engagement. The ability to see participants’ facial expressions can help the instructor to better assess the learning climate; however, it is can be challenging to assess learner comprehension by facial expression alone. When asking questions, inform your participants that you may call on them directly by name to alleviate uncertainty in who should be answering and prevent a single participant from dominating the discussion. If opening questions to the group at large, ensure that participants identify themselves prior to providing their response. Allow for longer pauses in between lines of questioning so participants have time to unmute their microphones.

Additional features available in the virtual learning platform can be used to further promote engagement. Audience response systems, such as polling and other modalities, offer immediate assessment of a learner’s acquired knowledge. If a polling feature is not embedded, consider using a chat feature where participants can type their responses. Provide high yield summations and encourage participants to use features such as “raise hand” or “thumbs up/thumbs down” to gage if there are questions or if learners are prepared to continue to the next concept. If appropriate, have some fun using games; utilizing the breakout room feature can facilitate team division and allow participants to engage in friendly competition.

## Tip 5: Focus on learner-centered goals and objectives

Learner-centered goals and objectives provide a fulcrum to ensure the outcome of learning is achieved and focus on the learner rather than technology. Avoid becoming consumed with technical functionality; rather, select modalities that promote attainment of the designated goals and objectives. Goals should provide a generalized landscape of content for the learning session, whereas objectives should be specific, measurable, achievable, relevant, and timely. Focusing on goals and objectives will keep learners on track with expectations and can help instructors adapt and modify content if goals and objectives are not able to be met in a given session.

## Tip 6: Optimize active learning

It is easy to fill a virtual learning system with dozens of articles and hours of videos while overlooking opportunities for active learning. Passive cognitive learning happens when a learner is solely tasked with observation; this occurs when reading an article, attending a lecture, or watching a video. This is less effective than active learning, where the learner is asked to engage, apply their knowledge to different circumstances and contexts, and reflect ( Graffam, 2007; Berkhout *et al*., 2017). Passive learning is best delivered asynchronously, by assigning readings or videos to be completed before a virtual learning session. In the “synchronous” virtual learning environment, active learning is best achieved through interactive and reflective exercises such as the use of case- or problem-based discussions or quizzes. Discussion boards can also create active learning; they should be task focused and engage students in a common purpose ( Graham *et al*., 2001). Instructors should attempt to have 25% of their content be interactive, weaving such elements throughout a session ( Simonson and Schlosser, 2004).

## Tip 7: Consider cognitive load

It is tempting to think that more is better; however, it is critical to recognize how cognitive load can impact the transition to virtual learning. Instructors should aim to balance the duration of a session with the quantity of content delivered to avoid learner fatigue. Prioritization of learner knowledge gaps can help instructors “select and plan for learning” ( Cutrer *et al.*, 2017), and learner centered objectives can provide an anchor for the goals to be obtained. Technology can enhance the delivery of education; however, it does not necessarily make learning faster. Make use of interactive exercises to cement key concepts and incorporate breaks throughout longer sessions so that learners can refuel. Communicate planned for breaks in advance to encourage engagement until a known opportunity to step away from the screen.

## Tip 8: Incorporate cooperative group learning

People learn more when working in groups than as individuals in many situations, though there is some debate as to whether this benefit is a result of the groups themselves or the chosen instructional methods ( Michael, 2006; Annand, 2011; Berkhout *et al.*, 2017). Emphasizing and practicing teamwork is especially important in medical education because of the significance of interpersonal skills. The acquisition of interpersonal skills becomes paramount when considering the inorganic nature of virtual learning. Cooperative learning takes many forms in an online learning environment. Using a blended learning strategy can promote cooperative learning with asynchronous pre-assignments and work presented when the group is together in a synchronous session. Group learning can be especially advantageous in large classes because, with careful planning, peers can help one another articulate understanding of the subject material and ask and answer questions in place of the instructor ( Michael, 2006). Group learning is also particularly useful for longitudinal curricular elements, for example working in teams to develop differential diagnoses from a clinical case presentation or treatment plans in an evidence-based fashion ( Chickering and Gamson, 1987; Graham *et al.*, 2001; Maggio *et al.*, 2018).

## Tip 9: Provide learners with meaningful feedback

Feedback contributes to learning by guiding a learner’s professional development ( Ivers *et al.*, 2012; Kornegay *et al.*, 2017). While many articles in the medical literature discuss how to promote feedback aimed at professional development, few consider how these lessons apply to virtual learning ( Kornegay *et al.*, 2017; Ramani *et al.*, 2019; Natesan *et al.*, 2019; Bonnel and Boehm, 2011). Strategies for successful face-to-face feedback may not directly translate to virtual learning. Online feedback is more likely to be asynchronous, and lack important non-verbal cues including posture, facial expressions, hand gestures, and tone of voice. Acknowledgement feedback in particular is often forgotten in the virtual environment as this normally happens almost unconsciously with eye contact or a nod in a face-to-face encounter.

Several different technologies can be used to provide feedback in virtual learning, including written, audio or video responses, pre-programmed automatic replies, and live web-based conference meetings. Rubrics provide a mechanism for assessing knowledge and are especially useful when working with a large number of learners ( Bonnel and Boehm, 2011). Peer-mediated and small-group feedback are other strategies that can be effectively used to provide feedback to many learners. Finally, it is important to be proactive about providing feedback in virtual learning. Feedback strategies should be determined prior to the sessions, as should be scheduled in advance to ensure that feedback is shared in a timely fashion. Properly utilized feedback methodologies promote professional development by helping learners identify areas of success, strive for improvement, and build an educational alliance with the instructor.

## Tip 10: Allow learners time to reflect

Reflection is an essential element of experiential learning - the process by which learning occurs through experience - and has been shown to empower deep cognitive processing and learning ( Sandars, 2009; Winkel *et al.*, 2017). Reflection can be defined as, “the process that occurs before, during and after situations with the purpose of developing greater understanding of both the self and the situation so that future encounters with the situation are informed from previous encounters” ( Sandars, 2009). Ideally, the learner’s reflection extrapolates the relevance of the topic beyond the current experience.

Technology allows for students to share their reflections in novel ways, including discussion boards, e-mail, and video. Additionally, virtual learning allows for creativity when designing opportunities for reflection. Instructors can trigger reflection by asking learners to answer thought provoking prompts throughout the learning session, or afterwards in a journal or discussion board. These prompts should aim to make the learner notice (“what surprised you in this case?”), process (“what does this mean?”) or consider future actions (“what could be done differently next time?”) ( Ménard and Ratnapalan, 2013). Collaborative peer discussions can also be used to promote reflection by requiring learners to think out loud, question their beliefs, and find new meaning. Summarizing, through creation of concepts maps for example, is yet another powerful tool for reflection ( Daley, During and Torres, 2016). No matter what reflective exercise is chosen, it should always be in synergy with the course’s learning objectives.

## Tip 11: Acknowledge instructors’ need to reflect

Instructors should likewise practice ongoing reflection. Reflection should occur “before, during, and after” delivery of an educational activity ( Cutrer *et al.*, 2017; Sandars, 2009). Instructors can engage in reflective practice either individually, or as part of a faculty development group ( Maggio *et al.*, 2018). Either way, reflection is especially important for instructors new to online education who often struggle to adapt effective virtual teaching strategies in place of their dominant teaching identity that has been primarily formed in more traditional settings ( Maggio *et al.*, 2018). The process of active reflection also allows instructors to inform future sessions by identifying the elements that are effective, and those that need improvement ( Cutrer *et al.*, 2017).

## Tip 12: Incorporate ongoing faculty development

Faculty development is an essential support system for educators adapting to their new virtual responsibilities. Faculty development endeavors have been shown to aid in the acquisition of new knowledge and skills, influence attitudes and behaviors, and potentially lead to changes in organizational practice ( Leslie *et al.*, 2013). Dedication to faculty development can help instructors identify mechanisms to enhance or modify curricular content and can serve as means for collaboration. Curriculum design, assessment, instructional design, and feedback are particularly important topics for educators entering the world of virtual education.

## Conclusion

When moving toward a virtual platform for education, consider the fundamental elements that shaped the activity if it were to remain “live”/in person. Technology should be leveraged as a tool but not detract from the instructor’s teaching identity or curricular content. No matter the format, instructional design should be effective, efficient, and appealing ( Merrill *et al.*, 1996). Incorporation of learner-based objectives, activities to promote learner engagement, and reflective practice will help to build learning environments and educational sessions that can withstand the physical distance.

## Take Home Messages

• Remember to consider the fundamental elements that defined the educational activity when it was to be conducted in person when designing a virtual session
• Technology is a tool to augment your educational strategies - it should not become a distraction from your teaching identity or curricular content
• Faculty development is essential to success
• Incorporation of learner-based objectives, activities to promote learner engagement and reflective practice are keys to success no matter what the physical distance

## Notes On Contributors

**Dr. Kheyandra Lewis** is an Assistant Professor of Pediatrics at Drexel University College of Medicine, an Associate Program Director for the pediatric residency and a pediatric hospitalist at St. Christopher’s Hospital for Children. She serves on the Implementation and Simulation Committees for the I-PASS SCORE Study Group.

**Dr. Zia Bismilla** is Assistant Professor of Pediatrics and hospitalist in the Department of Pediatrics at University of Toronto/Hospital for Sick Children. She is Director of Education in the Division of Pediatric Medicine. She serves on the Education and Training Committee for the I-PASS SCORE Study Group.

**Dr. Nicholas Kuzma** is an Assistant Professor of Pediatrics at Drexel University College of Medicine, the Pediatric Clerkship Director for DUCOM, and a pediatric hospitalist and Director of Educational Technologies at St. Christopher’s Hospital for Children. He serves on the Education and Training Committee for the I-PASS SCORE Study Group. He has led numerous didactics and workshops using various web conferencing technologies, and created several curricula using virtual learning platforms.

**Dr. Jennifer K. O’Toole** is anAssociate Professor of Pediatrics and Internal Medicine, and the Program Director, Internal Medicine-Pediatrics Residency Program, University of Cincinnati College of Medicine and Cincinnati Children’s Hospital Medical Center. She is the co-chair of the Education and Training Committee for the I-PASS SCORE Study Group and has extensive experience in curriculum design.

**Dr. Sharon Calaman** is a Professor (Clinical) of Pediatrics at NYU School of Medicine and an Attending in Pediatric Critical Care Medicine at NYU Langone Health. She has served as faculty for an online master’s program, and co-chairs the Education and Training Committee for the I-PASS SCORE Study Group.

## Declarations

Dr. Sharon Calaman and Dr. Jennifer K. O’Toole hold stock options and have done consulting work for the I-PASS Patient Safety Institute. The I-PASS Patient Safety Institute is a company that seeks to train institutions in best handoff practices and aid in their implementation. None of the other authors have any disclosures
