# Supplementary material for: Twelve Tips for Teaching with Virtual Learning Platforms
Source: MedEdPublish (2016). 2021 Mar 16;10:72. [Version 1] doi: 10.15694/mep.2021.000072.1 (PMC10939635; doi:10.15694/mep.2021.000072.1)
Supplement: Peer review history

# Reviewer report: Reviewer response for version 1

**DOI:** 10.21956/mep.18919.r27043

**Published:** 2021-06-12

**Authors:** Ken Masters

**Affiliations:** 1 Sultan Qaboos University

This review has been migrated. The reviewer awarded 3 stars out of 5 The article offers 12 tips for teaching with virtual learning platforms.The abstract should give a summary of the tips offered in the paper (Perhaps removed from the “Box” in the paper).Several of the early tips are not easily supported by the literature, and should be.The major problem with the paper, unfortunately, is that it has been over-taken by the wealth of similar papers published in the past year. If the paper had been published in March 2020, (perhaps even as late as July 2020), it would still have been novel. Published in March 2021, however, it, unfortunately, adds very little new insight to the field. Coupled with this, many of the tips are very broad, and there is little indication of how to implement them. For example, a novice (the person who would benefit most from a 12-tips paper) reads these sentences: “Discussion boards can also create active learning; they should be task focused and engage students in a common purpose (Graham et al., 2001). Instructors should attempt to have 25% of their content be interactive, weaving such elements throughout a session (Simonson and Schlosser, 2004).” The question is: “How?” These sentences, by themselves, offer very little guidance, and it would have been better if the authors had focused on one or two aspects, and then expanded on the How (An entire 12-tips paper could be written on the use of discussion boards, and that would certainly help the novice).So, ultimately, I think that the paper has been disadvantaged by timing, and, by the time it was published, the medical education field had moved on from the time when such a broad paper would have been useful. For future papers of this type, it would be useful if the authors could drill down deeply into specific aspects of online education, and focus on one or two specific areas.

Reviewer Expertise:

NA

No decision status is available

# Reviewer report: Reviewer response for version 1

**DOI:** 10.21956/mep.18919.r27044

**Published:** 2021-04-08

**Authors:** Rajaei Sharma

**Affiliations:** 1 College of Medicine and Health, University of Exeter

This review has been migrated. The reviewer awarded 4 stars out of 5 This piece addresses a vitally important area of medical education and is a great resource for medical educators. I appreciate the way in which there is acknowledgement of the pandemic as a causal factor but its continued bearing on education is not overstated. Many of the changes seen in medical education are likely to long outlive the pandemic and this piece begins to help that onward development. The entire piece is very well written but authors should remember that this online journal has a diverse global readership and so the language should be suitable for all. This is especially key for the abstract and the take home messages. It may also be beneficial to list some or all of the tips in the abstract so that it is a synopsis as opposed to a short introduction.The introduction clearly sets out the background and the basis for the piece. As the authors are writing from their collective experience, it may be additive to include some examples or more detailed discussion when referencing the recent transition to virtual platforms. “Consider the tips as a BOX” is confusing. The tips are all valuable and pertinent. Tips 1 and 2 especially come across quite prescriptive so could be adapted to be a little more discussive with examples or posed questions. Tip 5 could be expanded on (again remembering the global audience here) with what is meant be learner-centered goals; could perhaps compare and contrast with other styles of teaching. Tip 7 – brilliant and key to include. Tip 12 feels a little like an afterthought – worth expanding (especially as it is then referenced as one of the take home messages).Meaningful feedback for instructors is also a really important area here that should be included. Perhaps this was the intention with tip 12, but it is worth a stronger focus. Overall, this is a great piece that is well written and provides a comprehensive resource for educators looking to develop their virtual delivery. All of the points made here are with the intent of pushing this to the highest standard.

Reviewer Expertise:

NA

No decision status is available

# Reviewer report: Reviewer response for version 1

**DOI:** 10.21956/mep.18919.r27045

**Published:** 2021-03-17

**Authors:** BALAJI ARUMUGAM

**Affiliations:** 1 TAGORE MEDICAL COLLEGE AND HOSPITAL

This review has been migrated. The reviewer awarded 4 stars out of 5 Good written tips for improving virtual learning platforms. The most liked and interesting part would be Set ground rules for the session under the session etiquette is marvelous which speaks about every important aspect to be maintained during the initiation of any virtual learning sessions but surprisingly most of the learners, students or the faculty members those attend the program are not following properly in my experience in spite of setting the rules for the sessions. Either they remain dormant or create unnecessary noises during the progress of the sessions. Tip number – 3 Instructors to create or build a sense of community usually we call it as set induction or the ice breakers in pedagogy sessions which is again an important task for the instructors. Tip number 6 and 7 – considering the Cognitive load and the learner fatigue – to enhance the active learning especially in the virtual platforms to be addressed and it was well explained in this article. Thanks for the article on the tips for virtual learning platforms

Reviewer Expertise:

NA

No decision status is available
